# Temporal signatures of prediction error reveal external forces on an embodied robot

**DOI:** 10.3389/fnbot.2026.1918275

**Published:** 2026-10-14

**Authors:** Pierre Klintefors, Birger Johansson, Christian Balkenius

**Affiliations:** Lund University Cognitive Robotics Lab, Department of Philosophy and Cognitive Science, Lund University, Lund, Sweden

**Keywords:** cognitive robotics, humanoid, perturbation classification, prediction error dynamics, predictive processing, proprioception

## Abstract

Embodied robots must distinguish self-generated sensory consequences from externally caused perturbations. In predictive processing, such deviation is accounted for as prediction error, but it is commonly treated primarily as a signal to be minimised. Here, we test whether the temporal profile of load-related prediction error provides diagnostic information about the type of physical interaction a robot experiences. We trained and evaluated windowed multilayer perceptrons to predict servo current, used as a proxy for joint load, from proprioceptive, inertial, and servo current history. The training data were recorded during random movements of tilt and pan servo motors of a robotic head. The best model was deployed in the robot in real time and evaluated across free movements and external perturbations, including brief pushes, sustained forces, and obstacle interactions. Prediction error and raw current time series were summarised using features capturing magnitude statistics, temporal structure, and signal shape. A random forest classifier was then used to classify the conditions based on the features. Different interaction conditions produced distinct profiles that supported robust perturbation classification with high cross-validated accuracy. Classification performance for prediction error was equal to the classification of raw current. The results suggest that load-related prediction error is not only a residual of imperfect prediction. Its temporal structure can act as an embodied self-monitoring signal for classifying physical interactions.

## 1 Introduction

In this paper, we argue that prediction error signals of load-related force can be utilised to gather information about the environment and different types of external perturbation. A prediction error of force is not only a scalar deviation from the expected signal but also contains more useful information. Its temporal morphology, such as the onset, direction, peak, persistence, decay, cumulative bias, and phase relative to movement, can encode the physical cause of the discrepancy. The temporal dynamics of the error signal can thereby be used to infer body-environment interactions.

Due to the latency of sensory signals, an anticipatory mechanism is needed for efficient processing and real-time actions (Cameron et al., 2014). This argument is grounded in contemporary theories of sensorimotor control that emphasise prediction as the core feature (Seth, 2014). In predictive processing and active inference, perception and action are unified under the minimisation of prediction error (Adams et al., 2013; Friston et al., 2010). The frameworks do not distinguish between motor planning and sensory processing, since both utilise the same mechanism to close the gap between anticipated and registered sensory information. Sensory input is most informative when it diverges from internal model predictions, forcing either an update of the model or actions to change sensory inputs and resolve the discrepancy. In this paper, we investigate how the prediction error of servo current during motion can provide information about external obstacles and different types of perturbations. We want to test to what extent proprioception and joint load, in the absence of visual and tactile information, can be used for this perturbation classification.

Using a discrepancy between expected and observed joint-load-related signals is not new in robotics. Residual-based collision monitoring uses deviations between expected and observed robot dynamics to detect external contact (Haddadin et al., 2017; De Luca et al., 2006). For example, De Luca and Mattone (2005) derived a residual analytically from generalised momentum, allowing external collisions to be detected and further characterised without additional force sensors. Machine-learning approaches have also used temporal and non-linear features of proprioceptive contact signals to classify different forms of physical interaction. Golz et al. (2015) classified intended interactions and collisions from joint-torque profiles using a non-linear support vector machine. Our focus differs in that we investigate the temporal information contained in a prediction error signal itself and what the dynamics of the discrepancy between expected and observed load can reveal about the underlying body-environment interaction. We approach this question through predictive processing, where prediction error plays a central role in both sensorimotor regulation and learning.

The predictive processing perspective we adopt has clear historical roots. von Helmholtz (1948) described perception as unconscious statistical inference, emphasising recurrent loops of top-down and bottom-up signals rather than purely bottom-up representations that later drive action. In cybernetics, organisms are described as feedback systems that reduce discrepancies between the desired and actual conditions (Wiener, 1948). In ideomotor theory, actions are represented in terms of their anticipated sensory consequences, making behaviour inherently prediction-driven (Moeller and Pfister, 2022), a view that traces back to the 19th century (Carpenter, 1852). At its core, the ideomotor approach posits that motor control is not a process of commanding specific forces or positions, but rather a process of regulating an agent's dynamic relationship with its environment. This regulatory process is captured by impedance control, where mechanical impedance is defined as the ratio of force to velocity (Hogan, 1984). Further, active inference builds upon the concepts of enactment and agency, which are grounded in the ideas of embodied cognition, where cognition is always situated in a body interacting with the environment (Varela et al., 2017).

In line with these frameworks, we employ our in-house robotic platform, Epi (Johansson et al., 2020), to implement a prediction-error-based perturbation-detection and classification protocol in an embodied system. We focus on how the temporal dynamics of load-related prediction errors can be exploited to infer externally induced perturbations. If different types of perturbations can be associated with distinct error profiles, they can be used to guide a robot's behaviour.

The brain has been suggested to be in a constant process of inference, trying to find the best explanation for the stream of sensory information (Friston et al., 2010). The generative process that produces the sensory information is not directly accessible to the brain, but is inferred from an internal generative model. Under the active inference framework, movement is viewed as a set of proprioceptive predictions rather than explicit motor commands (Adams et al., 2013). Predictions descend from higher cortical areas, while muscle contraction is triggered at the spinal level to match the predicted sensory consequences. Accordingly, the central nervous system lacks a motor-sensory division and is a unified information system. There is a hierarchical structure to the generative model, where lower spinal reflexes can rapidly adjust to prediction errors and higher levels infer whether they are external or self-generated (Parr et al., 2019).

When it comes to motor learning, the generative model is built on the statistical regularities of proprioceptive signals, integrated with other sensor modalities. During early development, infants engage in “motor babbling,” a process where they explore the action space of their limbs by producing spontaneous, variable movements (Einspieler et al., 2008). A generative model of sensorimotor actions then emerges from this epistemic motor exploration, linking the movement with proprioceptive information. This dynamic way of motor control learning stands in contrast to the manually constructed forward and inverse kinematic models frequently used in traditional robotics (Nguyen-Tuong and Peters, 2011). However, a growing body of work in robotics has started to utilise motor babbling to learn and dynamically construct kinematic models (Aoki et al., 2016; Kase et al., 2022; Takahashi et al., 2017).

Multimodal information enriches a generative model, making it better equipped to generate versatile predictions in different contexts (Vasco et al., 2022). However, the inherent multimodality of human sensation makes it difficult to disentangle each sensory channel's individual contribution to force regulation. By using our robotic platform with a constrained sensorium, we can address the disentanglement issue. We test the ability to detect and classify external disturbances with limited sensory feedback using force prediction.

In predictive processing, descending projections from higher-order to lower-order areas are commonly associated with predictions, whereas ascending pathways convey sensory-driven signals, interpreted as prediction errors (Bastos et al., 2012; Feldman and Friston, 2010). The exact ratio of these pathways remains unclear, but anatomical studies consistently report that descending projections outnumber ascending ones in several animal models (Temereanca and Simons, 2004; Yang et al., 2025; Sermet et al., 2019; Markov et al., 2013). However, their functional influence is not solely determined by projection counts, but also by synaptic efficacy and context-dependent weighting (Temereanca and Simons, 2004).

Within predictive processing frameworks, this weighting is the estimated inverse variance of the noise of the generative process and the sensory states (Friston et al., 2010). For an accurate model, the agent needs to learn to distinguish between generic noise and self-generated noise caused through action (Parr et al., 2019). This relates to the documented effect of sensory attenuation where stimuli generated by voluntary movement result in a weaker neurophysiological response than external sources (Hughes et al., 2013). Attention and signal variance can then modulate the relative influence of top-down predictions and bottom-up sensory signals (Feldman and Friston, 2010; Proietti et al., 2025). An analogous principle exists in control theory in the context of state estimation. For example, a Kalman filter combines a prior prediction with a sensory measurement through an uncertainty-dependent gain term *K*, effectively weighting their relative contributions (Kalman, 1960).

Using internal predictions enables the system to generate rapid estimations and a smooth signal. By continuously grounding those predictions to incoming sensory evidence, drifting is avoided and stability is maintained without noisy fluctuations. The precision update in predictive processing can therefore be interpreted as an uncertainty-weighted state-updating process, with a trade-off between speed and accuracy.

When embracing predictive processing, the motion trajectory and the applied force that realises that trajectory are both part of the same process of prediction error minimisation (Friston et al., 2010; Adams et al., 2013). Rather than planning force and motion independently, the two are coupled through dynamic primitives: attractor-like patterns in the state space that generate coordinated trajectories and corresponding forces (Hogan and Sternad, 2012). This principle is demonstrated in biological systems. Humans rely on an integrated somatosensory feedback system to detect and adapt to externally caused perturbations, combining proprioceptive signals from muscle spindles and Golgi tendon organs with tactile input from cutaneous mechanoreceptors (Jayasinghe et al., 2021).

While tactile and visual feedback improve precision in movement tasks such as grasping (Carteron et al., 2016), proprioception alone can sustain adaptive adjustments in force control. This was demonstrated by Mugge et al. (2012), who showed that participants could adjust the force of their ankle in the absence of tactile or visual signals. Proprioceptive signals from muscle spindles are particularly suited for rapid force corrections because of their low latency (Dimitriou, 2022). Furthermore, Cheng et al. (2025) suggest that force regulation, in terms of impedance control, can be understood as a mechanism for modulating visuo-haptic information. They observed that muscle coactivation increased with haptic noise and decreased with visual noise. Limbs become stiffer when haptic noise increases, whereas they relax when visual noise increases. In this way, impedance is adjusted to rebalance reliance on visual and haptic information. This active sensing strategy highlights a crucial trade-off between perceptual benefit and effort.

Our work builds on these biological and theoretical insights by investigating to what extent information from proprioception and actuator current can enable an embodied agent to infer and classify externally caused perturbations. The robotic platform we use has limited sensorium, mimicking the proprioceptive information from muscle spindles via servo motor position and current, and vestibular information from an onboard Inertial Measurement Unit (IMU). The absence of tactile feedback is a key feature of our experimental design, allowing us to isolate and study the contributions of proprioceptive and inertial data to the process of inferring physical interactions.

Our study demonstrates an embodied predictive self-monitoring mechanism in which a learned sensorimotor forward model produces load-related prediction errors whose temporal structure supports classification of external physical interactions. We trained several neural networks on proprioceptive and servo current history, with or without accompanying inertial data, to predict the next-step servo current. The network serves as the system's internal generative model, making feedforward predictions about the expected servo current based on the proprioceptive and inertial states. The dynamics of the prediction errors then serve as a central and informative variable. We hypothesise that the temporal profiles of these servo current prediction errors can be used to classify different perturbation categories. This mechanism aligns with the principles of active inference (Parr et al., 2019). It provides a closed feedback loop that uses cybernetic error-driven regulation and the ideomotor concept of action through anticipated sensory effects. We believe that this study illustrates the importance of physical situated sensory motor learning and error-based interaction monitoring. By processing the temporal information in an error signal, a system becomes more adaptive and versatile in utilising the available resources of its sensorium.

## 2 Materials and methods

To investigate how the temporal structure of load-related prediction errors encodes information about external perturbations, we designed an experiment using a robotic platform with a constrained sensorium and a trained feedforward network serving as the internal generative model. Joint load was approximated from servo current, which served as the prediction target. This choice also improves practical availability, since no additional torque sensors are required. Prediction-error profiles were generated from controlled perturbation conditions and used to test whether different perturbation types produced distinguishable temporal error patterns.

### 2.1 Robotic platform

We implemented and tested the approach on our in-house robotic platform Epi (Johansson et al., 2020). Figure 1 shows the two-degree-of-freedom pan-tilt robotic Epi head actuated by Dynamixel MX-106R servomotors (ROBOTIS Co., Ltd., 2023). The pan servo is mapped to the horizontal axis and the tilt servo to the vertical axis, providing a controlled but sufficiently rich action space for generating varied motor transitions. Mounted inside the head (see Figure 1B) was a UM7 inertial measurement unit (IMU) that records accelerometer and gyroscope data (Redshift Labs, 2018).

**Figure 1:**
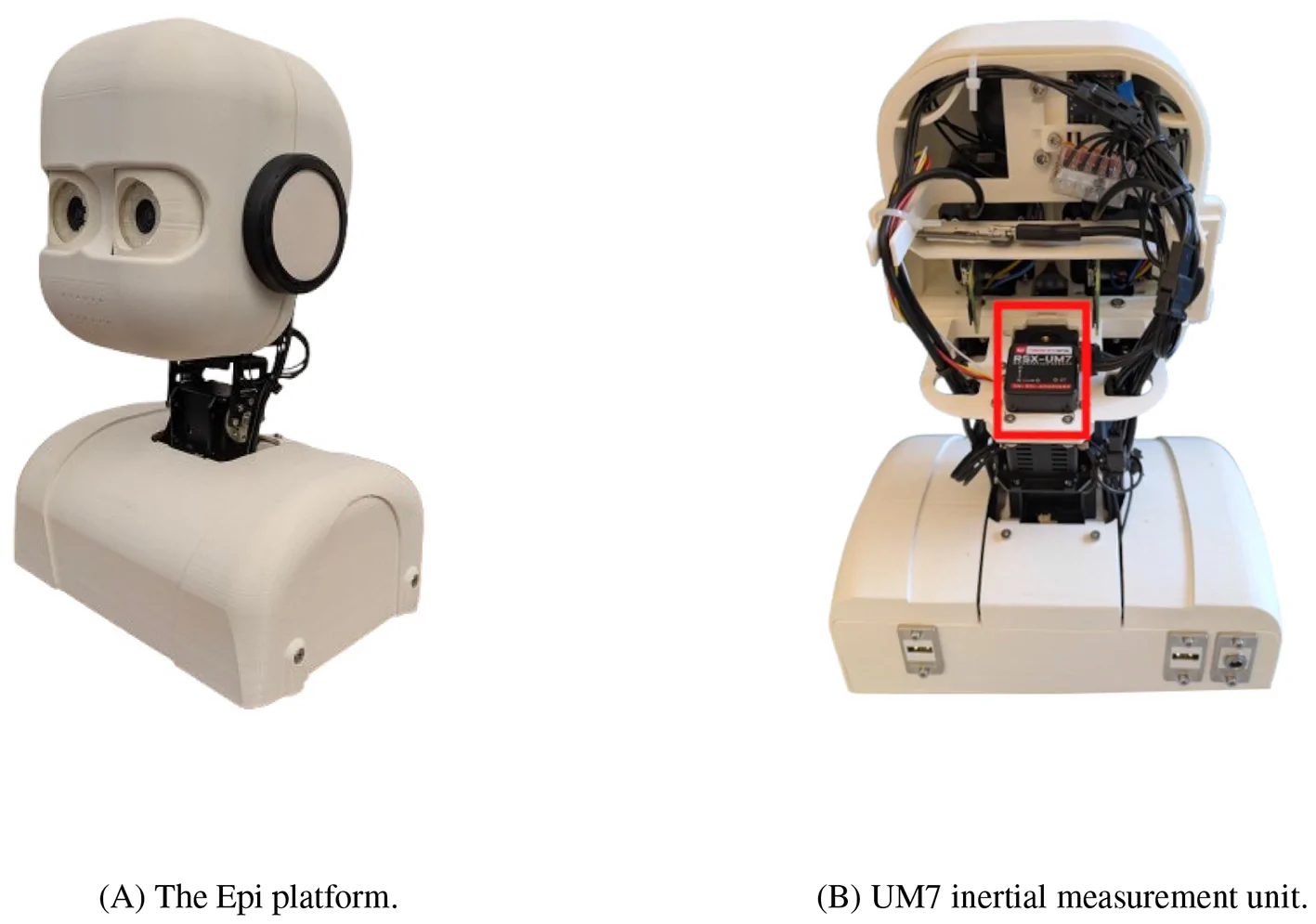
The robotic platform **(A)** and the inertial measurement unit mounted inside its head **(B)**. Only the robot's tilt and pan motors were used for the experiment, not the camera motors or cameras.

### 2.2 Testing infrastructure

Ikaros, our infrastructure for cognitive modelling, was used for the experiments with the Epi robot (Balkenius et al., 2020). Ikaros utilises a dataflow programming paradigm that enables parallel computation and modularisation of processes. Ikaros ran on C++ code, while the generated predictions ran as TensorFlow Lite models in Python v. 3.12 (Python Software Foundation, https://www.python.org/). Ikaros communicated with the Python scripts via shared memory for low latency inter-process communication (IPC). The dataflow is illustrated in Figure 2. During inference, the Ikaros infrastructure and TensorFlow models ran on a MacBook Pro with an Apple M2 chip and 8 GB of RAM. The proprioceptive and inertial data were sampled at 40 Hz, the same rate as the recorded training data, and fed into the network. This ensured consistency between the training and deployment conditions while enabling efficient real-time current prediction and perturbation detection.

**Figure 2:**
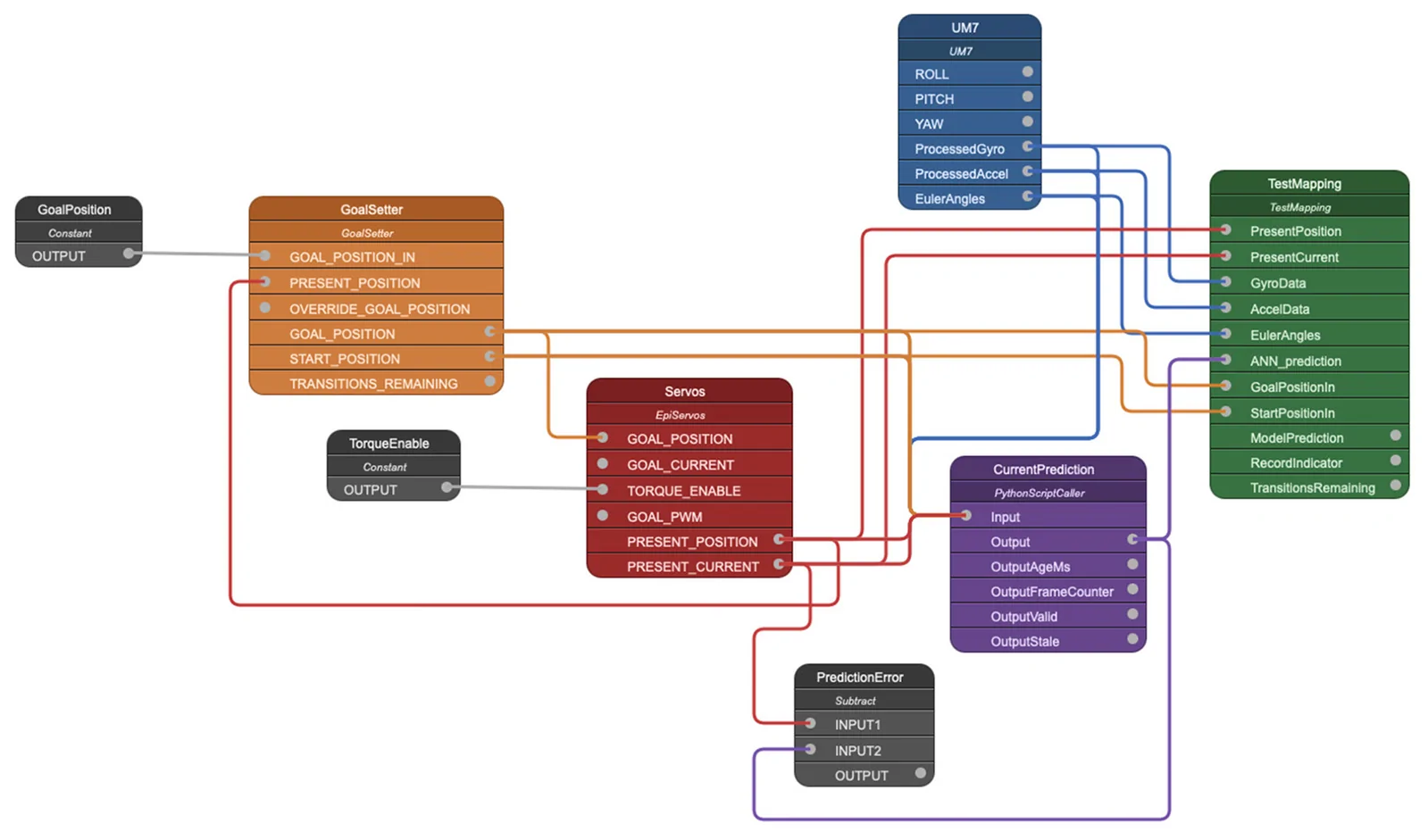
The connecting input and output of modules in the computational infrastructure Ikaros when testing prediction error dynamics of servo current in a robot. The colour of the connections matches the colour of the outputting module. The current prediction module in Ikaros communicates with Python scripts via shared memory.

### 2.3 Training and test data

The dataset consisted of 2,000 transitions of randomised servo positions, with a 500 ms pause between each transition. This resembles the process of motor babbling seen in infants when shaping the generative model. The tilt motor was constrained to the range 122°–237° and the pan motor to 130°–240°, where an upright neutral position corresponds with a set position of 180° in both servos.

During each transition, proprioceptive and inertial data were sampled at 40 Hz. The recorded variables included gyroscope values (*x, y, z*), accelerometer values (*x, y, z*), Euler angles (*x, y, z*), servo positions, distance from the start and goal positions, and servo current. For each model variant, a sequence of previous samples was used as input to predict the servo current at the next sampling step. The position and servo-current values were obtained directly from the Dynamixel MX-106R servos via the Dynamixel communication protocol. Servo current therefore refers to the current estimate reported by the servo firmware rather than an independently measured electrical current.

The dataset was split into training and test sets, with 80% used for training and 20% reserved for testing. The data were normalised prior to training. To capture temporal dependencies, the training and test sets were segmented into overlapping input sequences of varying temporal context lengths. This segmentation was performed separately after the train-test split to avoid data leakage.

### 2.4 Model architecture and training

The neural networks trained for prediction used a windowed multilayer perceptron (MLP) architecture. They were implemented in TensorFlow/Keras and served as the internal generative models. Each model received sequenced inputs consisting of consecutive samples of proprioceptive, inertial, and/or previous-current features, depending on the feature condition, and predicted the electric current of the two servos at the next time step. By holding the architecture constant while varying only the feature set and history lag, the model comparison was designed to evaluate the relative importance of available sensorimotor information and temporal context for predicting servo current. The networks were trained in a Google Colab environment using Google's TPUv4 hardware accelerators.

The network architecture is displayed in Figure 3 and consisted of four fully connected hidden layers with 256, 128, 64, and 32 units, respectively. The windowed MLP was selected as a deliberately simple fixed-context architecture that could be deployed straightforwardly using TensorFlow Lite while satisfying a real-time 40-Hz inference requirement. While recurrent architectures like GRUs or LSTMs inherently handle sequential data, their step-by-step recurrent execution introduces a sequential bottleneck can scale poorly during edge deployment. Similarly, 1D-CNNs incur data-shuffling memory overhead from sliding kernels. Unlike recurrent models, the windowed MLP does not maintain a recurrent hidden state, and the complete temporal window can be evaluated in a single feedforward pass. However, we did not systematically benchmark the computational efficiency of alternative recurrent or convolutional architectures, and therefore do not claim that the MLP is optimal in either predictive accuracy or inference efficiency.

**Figure 3:**
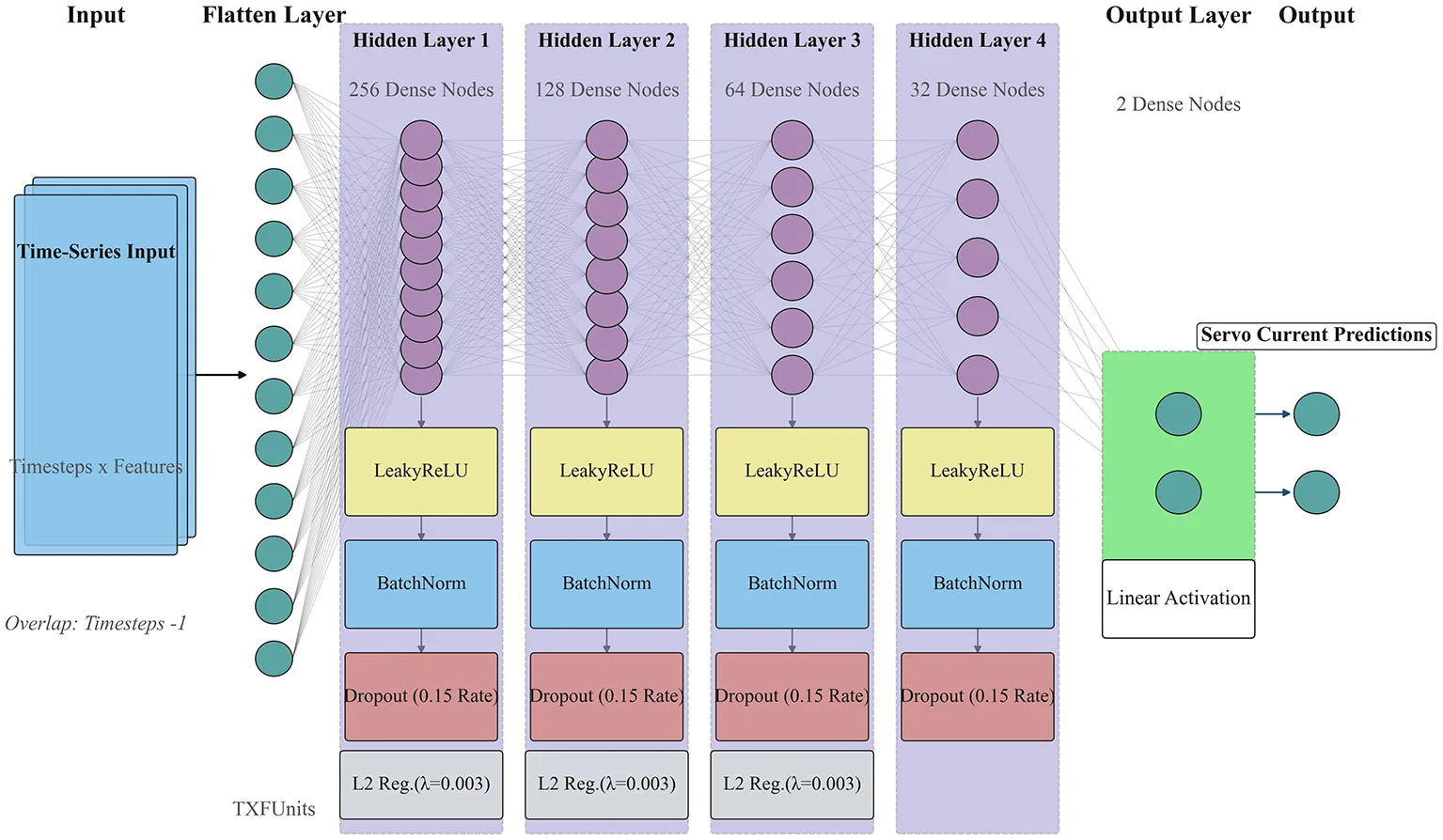
Architecture of the windowed multilayered perceptron trained to predict the servo current from the servos.

The network weights were initialised by the He-normal function. Each layer used a leaky rectified linear unit (LeakyReLU) activation function, L2 regularisation (λ = 0.003), and a dropout rate of 0.15. Batch normalisation layers were included after each hidden layer to stabilise learning. The output layer contained two linear nodes corresponding to the predicted servo currents. Training was conducted using the Adam optimiser (learning rate = 0.0001) and a Huber loss function (δ = 1.8) to ensure robustness against outliers. Early stopping with a patience of 25 epochs was employed to prevent overfitting, and training was run for a maximum of 400 epochs. The model was monitored using mean squared error (MSE) as a performance metric.

The network architecture and training parameters, including layer sizes, regularisation strength, dropout rate, learning rate, and Huber-loss parameter, were selected during an initial model-development phase. This hyperparameter selection was performed once rather than separately for each feature set or temporal-history condition. The resulting architecture and training parameters were subsequently held constant across all model comparisons to allow the effects of input features and temporal context to be evaluated under the same model configuration. Each of the 18 resulting configurations (three feature sets × six history-lag conditions) was trained three times using different random seeds, resulting in 54 final training runs. This was done to reduce dependence on the stochasticity of network initialisation and training. Reported MSE values are thereby the mean across the three runs for each configuration.

To evaluate informative input features, three different sets of input features were trained with the same windowed MLP architecture. One contained only positional information, one contained both positional and IMU data, and one contained positional information and previous servo current measurements. The features included in each set are displayed in Table 1. We varied the temporal context from 0 to 15 historical lags (0, 1, 3, 5, 10, and 15 lags). Because the contemporaneous sample was retained, these corresponded to input windows of 1, 2, 4, 6, 11, and 16 time steps, respectively.

**Table 1:** Input features used across the three windowed MLP configurations.

Feature	With IMU	No IMU	No IMU + Current
Tilt			
Tilt position	✓	✓	✓
Tilt distance to goal	✓	✓	✓
Tilt distance to start	✓	✓	✓
Tilt current	–	–	✓
Pan			
Pan position	✓	✓	✓
Pan distance to goal	✓	✓	✓
Pan distance to start	✓	✓	✓
Pan current	–	–	✓
IMU			
Gyro (X, Y, Z)	✓	–	–
Acceleration (X, Y, Z)	✓	–	–
Angle (X, Y, Z)	✓	–	–
**Total features**	**15**	**6**	**8**

The feature counts (15, 6, and 8) refer to the number of features at each time step. Models were evaluated using 0, 1, 3, 5, 10, or 15 historical lags. Because the contemporaneous input was retained, a model with *L* historical lags received *L*+1 time steps, giving a flattened input dimensionality of *F*(*L*+1), where *F* is the number of features per time step. Bold indicates total number of features.

### 2.5 Perturbation classification experiment

Once trained and evaluated, the chosen model for perturbation classification was converted to TensorFlow Lite (tf.lite) for real-time deployment. The tilt and pan motors were tested and classified independently across five conditions: (1) a brief manual push applied to the stationary head, (2) sustained manual force applied at trial onset and held throughout the trial, (3) a rigid obstacle mounted on an external structure that intercepted the head during a 30° movement, (4) an unperturbed 5° movement baseline, and (5) an unperturbed 30° movement baseline. Fifty trials of each perturbation and non-perturbation condition were conducted, where each trial lasted two seconds with a one-second inter-trial interval. The tilt and pan servo data used in the classification were from different trials, meaning 250 trials for each servo (50 trials × 5 conditions) with a total of 500 trials.

The two manual perturbation conditions were applied by hand with intentionally varied force amplitudes but a consistent direction. This allowed a range of push magnitudes for better representing the perturbation category rather than a single force level.

To compare the temporal structure of prediction errors across perturbation conditions, each trial-level error signal was transformed into a set of summary features capturing error magnitude, temporal distribution, persistence, and signal shape. This signal shape is targeted using amplitude-normalised features, such as discrete cosine transform (DCT) coefficients. The full set of extracted features is shown in Table 2. Classification was evaluated separately using magnitude features, amplitude-normalised temporal/shape features, the combined prediction-error feature set, and the corresponding features extracted from raw current.

**Table 2:** Features extracted from each trial's prediction error signal.

Feature	Definition
Baseline magnitude statistics	
Maximum absolute error	Largest absolute prediction error during the trial
Standard deviation	Standard deviation of *e*(*t*)
Mean error	Mean value of *e*(*t*) across the trial
Cumulative error	Sum of *e*(*t*) across the trial
Error range	Difference between the maximum and minimum error
Temporal structure and energy distribution	
Normalised phase-binned means	Normalised mean absolute error in five equally sized temporal bins, *M*_1_–*M*_5_
Early-to-late ratio	Ratio between mean absolute error in the first and final 30% of the trial
Time to peak	Normalised time point at which |*e*(*t*)| reaches its maximum
Half-decay time	Normalised time after the peak until |*e*(*t*)| falls and remains below 50% of its peak value
Lag-1 autocorrelation	Pearson correlation between *e*(*t*) and *e*(*t*+1)
Temporal centre of mass	Amplitude-weighted temporal centre of the absolute error signal
Sign consistency	Proportion of timesteps with the same sign as the peak error
Morphological shape coefficients	
DCT-II coefficients	First four AC coefficients (*c*_1_, *c*_2_, *c*_3_, *c*_4_) computed from the amplitude-normalised error signal

The feature vector consisted of 5 magnitude features, 11 temporal features, and 4 morphological shape coefficients.

#### 2.5.1 Classification, cross-validation, and statistical testing

To evaluate the separability of the five experimental conditions based on the extracted error-profile characteristics, a Random Forest classifier was employed. Features were standardised to zero mean and unit variance prior to classification. To estimate generalisation performance while preventing information leakage during hyperparameter optimisation, classification was evaluated using nested cross-validation. The tilt and pan servo was classified where their data did not originate from the same trial. The outer evaluation loop used five-fold (*K* = 5) stratified cross-validation, while hyperparameter optimisation was performed within each outer training set using four-fold (*K* = 4) stratified cross-validation. Tilt and pan data were collected in separate physical trials. No physical trial therefore contributed to both a tilt and a pan observation, and signals originating from the same trial could not appear in different cross-validation folds.

The inner grid search varied the number of estimators (100, 300, 500), maximum tree depth (3, 5, 7), and minimum number of samples required for splitting (2, 5, 10). After hyperparameter selection, the model was refitted on the complete training portion of the corresponding outer fold and evaluated on its held-out test portion. The same outer folds were used for all feature representations to enable direct comparison of classification performance. Overall accuracy was calculated from the combined out-of-fold predictions, such that each observation was evaluated only when held out from model fitting and hyperparameter optimisation.

To quantify uncertainty in classification performance, 95% confidence intervals were estimated using non-parametric bootstrap resampling (*B* = 10, 000). The out-of-fold predictions from the nested cross-validation were retained, and experimental groups were resampled with replacement. Classification accuracy was recalculated for each bootstrap sample, producing an empirical sampling distribution from which the 2.5th and 97.5th percentiles where used for 95% confidence intervals.

To determine whether classification performance exceeded that expected in the absence of a relationship between the extracted features and experimental condition, a permutation test was conducted (*N* = 100 permutations). The theoretical accuracy expected from uniform random guessing across the five classes was 20%. For each permutation, condition labels were randomly shuffled, thereby breaking the relationship between features and class labels while preserving the overall class frequencies. The complete nested cross-validation procedure, including inner-loop hyperparameter optimisation, was then repeated on the permuted dataset. This produced an empirical null distribution of cross-validated accuracy under the hypothesis that the extracted features contained no discriminative information about experimental condition. The empirical *p*-value was calculated according to Equation 1:


$$\begin{array}{l} p=\frac{\sum_{i=1}^{N}\text{𝕀}\left({\text{Acc}}_{\text{perm},i}\geq {\text{Acc}}_{\text{obs}}\right)+1}{N+1}, \end{array}$$ (1)

where *I*(·) is the indicator function, Acc_perm, *i*_ is the nested cross-validation accuracy obtained for the *i*th permuted dataset, and Acc_obs_ is the accuracy obtained using the original, unpermuted condition labels.

The classification analysis was used as a quantitative test of whether the experimentally observed prediction-error profiles were separable based on their temporal characteristics. The aim was not to identify an optimal classification architecture or feature representation, but to complement the descriptive trajectory analysis with an out-of-sample measure of condition separability.

## 3 Results

### 3.1 Neural network evaluation

Several windowed MLP models were trained with and without IMU features using different history lags (0, 1, 3, 5, 10, and 15). The results are shown in Figure 4. Models with IMU data outperformed those without IMU data when little or no temporal context was available (0 and 1). This difference diminished for models using longer input sequences (>3 steps).

**Figure 4:**
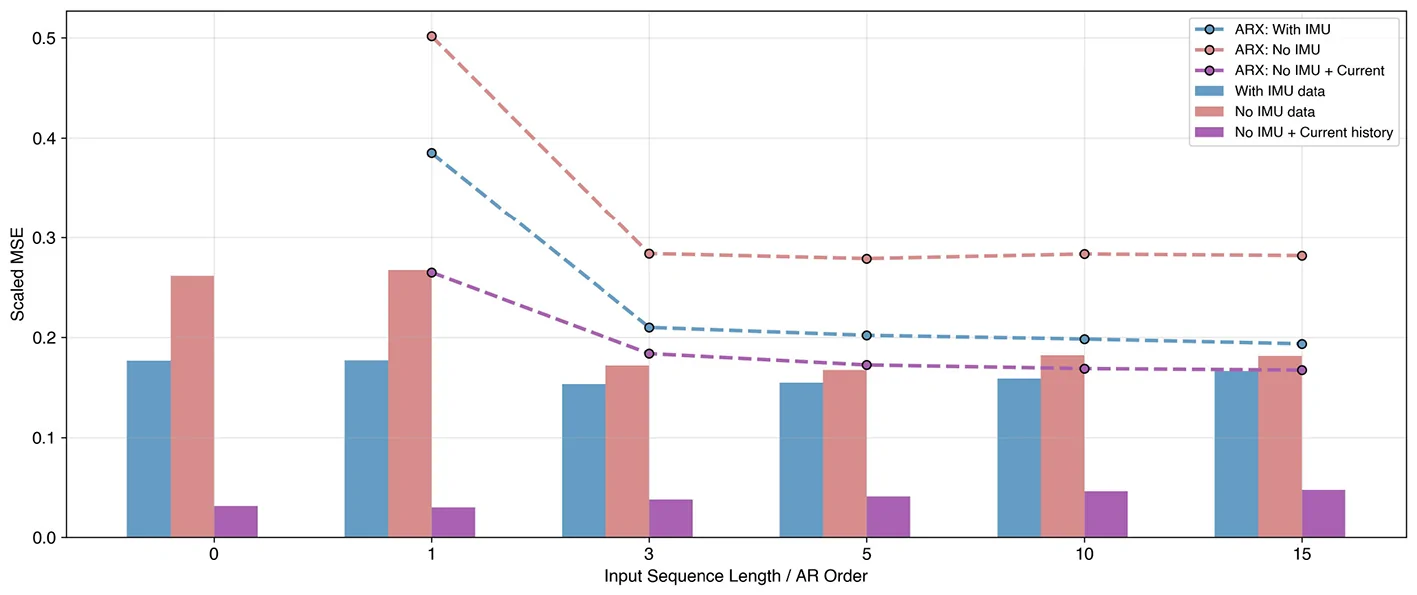
Mean squared error (MSE) for predicting motor current using multilayer perceptrons (MLPs) trained on windowed temporal input (mean of three training runs per configuration), across three feature sets: position only (No IMU), position and current history (No IMU + Current), and position with inertial measurement unit data (With IMU). Window sizes of 0, 1, 3, 5, 10, and 15 time steps were evaluated for each feature set. Coloured lines show matched linear ARX (autoregressive with exogenous input) baselines using the same lagged inputs and prediction target as each corresponding feature set, for window sizes ≥1. The order-0 ARX baseline (intercept-only, no predictors) is omitted from the plot as it is not directly comparable to the MLPs' window-0 condition, which retains contemporaneous features.

An additional set of temporal models, without IMU data but with servo current history as input features, was also trained. As shown in Figure 4, increasing the history lag did not improve the performance of these models beyond a single time step; one time step back (25 ms) was sufficient to reliably predict the next, and MSE remained essentially flat from 1 through 15.

To assess whether the windowed MLPs capture temporal structure beyond what a linear model can extract from the same inputs, we compared each MLP feature set against a matched autoregressive-with-exogenous-input (ARX) model using identical lagged predictors and prediction target (Figure 4). All three ARX baselines showed a similar pattern: a large drop in MSE from order 1 to order 3, followed by a plateau with negligible further improvement from additional lags. This mirrors the trend seen in the corresponding MLP models and indicates that, for both model classes, most of the exploitable temporal information is captured within the first few time steps.

Notably, every MLP feature set outperformed its matched ARX baseline at every history lag tested. This gap indicates that the low error achieved by the current-history MLP is not simply a consequence of a linearly relationship between current and future current values. The linear model given the same one-step history performs markedly worse, suggesting the MLP is exploiting nonlinear structure that the ARX baselines do not capture.

### 3.2 Perturbation classification

Based on the performance evaluation in Figure 4, the windowed MLP with servo current history as input was selected for the perturbation classification experiment. A temporal window of three history lags was chosen to enable the network to derive positional derivatives. To avoid drift in the prediction error signal, the current input to the network was a weighted blend of the predicted and actual servo current from the previous inference iteration, with 80% and 20% for predicted and actual current, respectively.

The average prediction errors (predicted-actual) are displayed in Figure 5. Negative prediction errors correspond to underestimations, while positive errors correspond to overestimations. Prediction errors were present for both the perturbed and non-perturbed trials. Furthermore, the different perturbation conditions displayed different prediction error profiles. A brief push was associated with a sharp peak that then stabilised within 400 ms. When sustained force was applied to the head, the error signal had a long tail. When a stationary obstacle intercepted the trajectory of the head, the signal had a higher amplitude than the same movement without the obstacle. The non-perturbed movements of 5° and 30° displayed similar error profiles, but the peaks of 30° movements had a larger amplitude.

**Figure 5:**
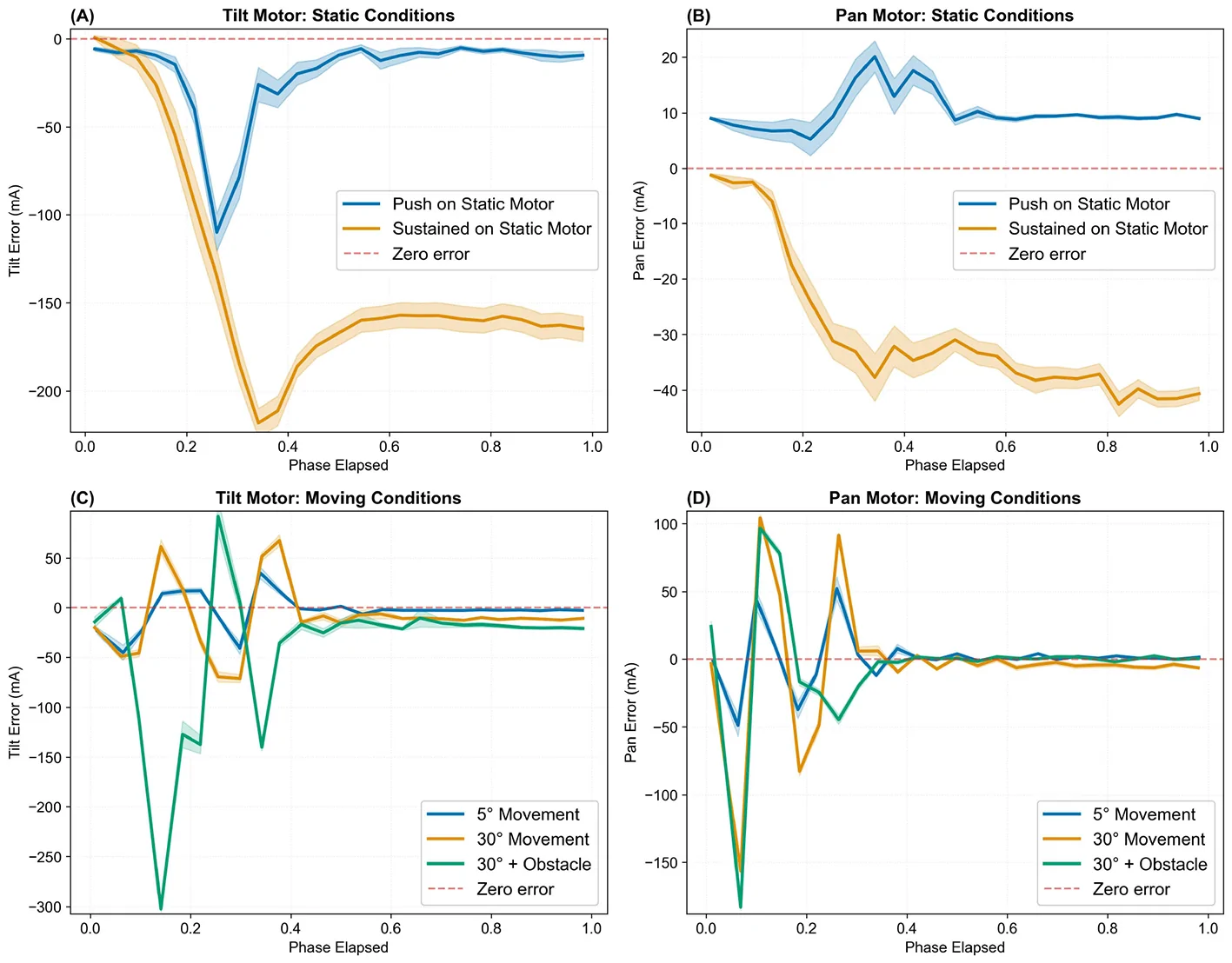
Descriptive graph of average prediction error signal across static and moving conditions for the tilt and pan motors. The error signal of all trials has been phase-aligned according to the start and end of each trial. Panels **(A, B)** show the static perturbation comparison between push and sustained conditions for the tilt and pan motor, respectively. Panels **(C, D)** show the comparison of moving conditions for 5° movement, 30° movement, and 30° movement with an obstacle, plotted on the same axes for each motor. Solid lines indicate the mean prediction error across phase and shaded regions denote ±1 standard error of the mean.

The normalised classification performance is displayed in the confusion matrix in Figure 6. Panel A of the figure shows classification using the prediction error of servo current, achieving high accuracy (≥98%) across conditions. The static sustained perturbation was misclassified as a push in 2% of trials, which was the most common misclassification. Feature importances were extracted from the random forest classifier to identify which features most strongly guided classification.

**Figure 6:**
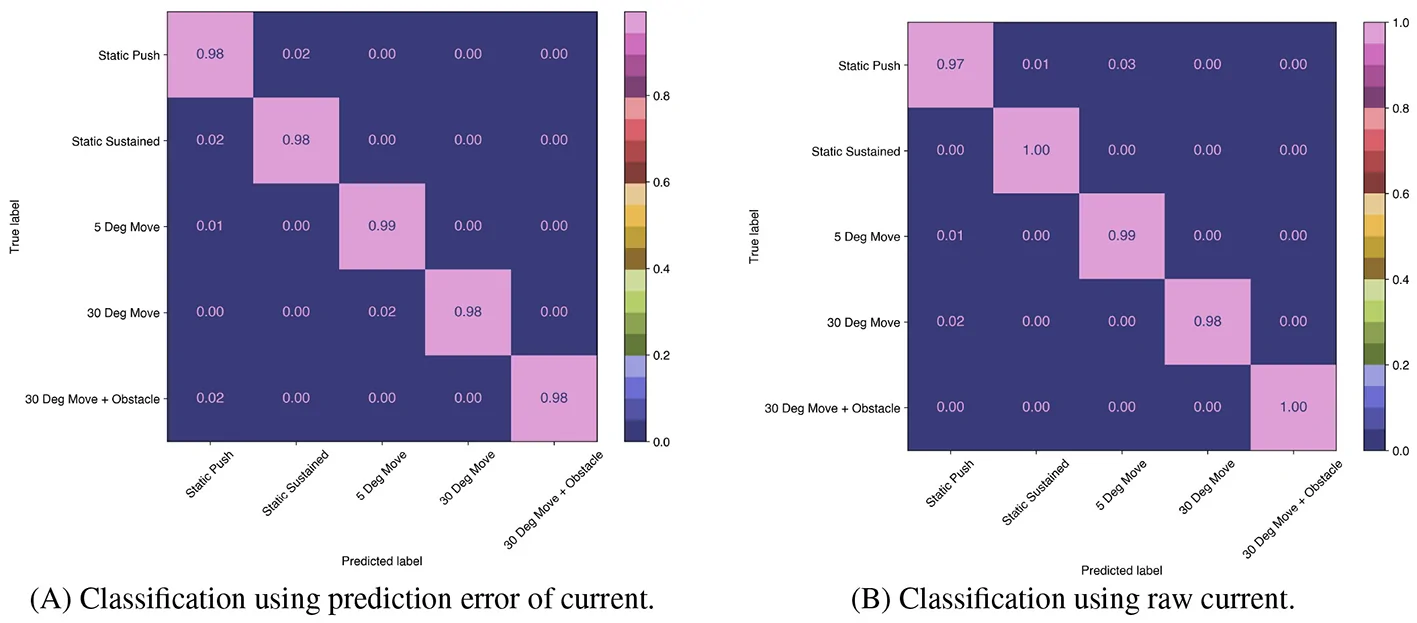
Normalised confusion matrices for the Random Forest classifier across the five interaction conditions. Values on the diagonal represent the proportion of correctly classified trials for that condition, evaluated using out-of-fold predictions. **(A)** shows classification using the prediction error between predicted and raw current, **(B)** shows the classification using the raw current.

Classification performance of the error signal across feature sets is summarised in Table 3 and Figure 6A. Magnitude features alone achieved an accuracy of 95.9 ± 2.3% (95% CI [94.0%, 97.6%]). Amplitude-normalised temporal and shape features had an accuracy of 97.6 ± 1.3% (95% CI [96.1%, 98.9%]), confirming that interaction conditions remain highly distinguishable even without magnitude information. Combining magnitude and temporal features achieved 98.5 ± 0.9% (95% CI [97.4%, 99.6%]).

**Table 3:** Cross-validation classification accuracy and 95% bootstrap confidence intervals across feature representations.

Feature set	CV accuracy (%)	95% CI (%)
Magnitude only	95.9	[94.0, 97.6]
Temporal/shape only	97.6	[96.1, 98.9]
All features (Error)	98.5	[97.4, 99.6]
All features (Raw current)	98.5	[97.2, 99.6]

A permutation test confirmed that classification accuracy significantly exceeded chance level (20.0%), with none of the 100 permuted iterations reaching the observed accuracy of 98.5% (null distribution mean: 18.8 ± 3.1%; *p* = 0.0099).

Feature importances are displayed in Table 4, where Temporal Centre of Mass (13.31%) and the first phase-bin mean ( 13.20%) were the most discriminative features. Finally, evaluating raw current directly resulted in an accuracy of 98.5 ± 0.9% (95% CI [97.2%, 99.6%], where Figure 6B displays the accuracy of individual conditions. The classifier was thereby as successful in classifying prediction error as the raw current.

**Table 4:** Feature importances of the random forest classification.

Feature	Importance (%)
Temporal Centre of Mass	13.31%
Bin Mean 1 of 5	13.20%
Max Absolute Error	7.45%
DCT Coefficient 1	7.18%
Range	6.84%
STD Error	6.48%
Early-to-Late Ratio	6.47%
Mean	4.94%
DCT Coefficient 4	4.93%
Sum Error	4.73%

## 4 Discussion

We evaluated how the temporal dynamics of a load-related error signal can be used to classify different types of external perturbations. The results show that proprioceptive information provides a system with substantial information when inferring and classifying perturbations. We trained and evaluated our models by deploying them on a physical robotic platform. This made the signal include real actuator dynamics, friction, inertia, and head-environment interaction. In this setting, we found support for our central hypothesis: that the temporal dynamics of a prediction error can be used to classify different kinds of external perturbations. This is the main contribution of the study: it shows that the temporal characteristics of the prediction-error signal are themselves sufficient to support high classification accuracy in the tested conditions, even after amplitude normalisation. We drew inspiration from theories of predictive processing, in which biological systems utilise prediction error for efficient processing and self-monitoring.

### 4.1 Network evaluation

The evaluation of networks with different sets of input features provided multiple insights. Firstly, the benefits of having IMU data about the inertial states disappeared when the history lag was at least three. Having temporal context with three time lags allows the network to implicitly learn local movement dynamics of the system by inferring velocity and acceleration without a specialised sensor. Since the robot was placed on a stable surface, the IMU angles did not provide any additional information beyond the servo positions.

In the human body, the vestibular system provides information about the head's inertial states, but other joints lack this information. However, storing proprioceptive history would allow the central nervous system to derive the velocity and acceleration of a limb or end effector. Such short-term proprioceptive memory can be accessed explicitly (Chiyohara et al., 2023), but an implicit cerebellar representation of trajectory history is sufficient (Kawato, 1999; Wolpert et al., 1998; Blakemore et al., 2001).

A second takeaway from the model comparison was that including previous servo current notably increased the network's predictive power. This is expected, as incorporating past values of the target variable effectively transforms the problem from a static regression task into a time-series forecasting problem. The central nervous system has access to information about previously generated force, both through motor neuron recruitment (Contessa et al., 2009) and feedback from the Golgi tendon organs (Moore, 1984). Thus, a control strategy that incorporates force history is biologically plausible and improved predictive accuracy in the present models.

### 4.2 Perturbation classification

The chosen classification method achieved an accuracy of ≥98%, suggesting that the tested interaction conditions produced reliably distinguishable error profiles under the present experimental conditions. This result should not be interpreted as evidence that the selected feature representation or Random Forest classifier is optimal. There were some minor misclassifications in our experiment, predominantly between a brief push and the sustained perturbation on a stationary head. These two had similar initial phases in their dynamic profiles with a sharp peak in current. There was also a small proportion of misclassifications of a brief push as a small 5° movement, since the initiation of movement was associated with a sharp peak in servo current. In biological systems, such ambiguities can be resolved by tactile information, where feedback signals can temporarily be dampened if they are consistent with the predicted action (Hughes et al., 2013).

In our system, the windowed MLP predicted the expected current from the movement and was not accurate enough to perfectly predict the servo current for a non-perturbed movement. Despite this, a simple random forest classifier was largely successful in distinguishing between these similar events, using recent history of proprioception and servo current. With more multimodal feedback, such as vision and tactile, a more sophisticated generative model can be established. For instance, while proprioceptive signals may be attenuated during movement, tactile feedback can increase in relative gain when contact occurs, allowing the system to remain sensitive to external events even during active motion.

Additionally, in our inference testing, the windowed MLP received information about the distance to the goal and the start position, but only in the context of predicting servo current. However, the same predictive architecture could be extended to support a more explicit sensory attenuation mechanism in a hierarchical control framework, where predicted sensory consequences are selectively down-weighted at higher processing levels. Such an extension would allow the system not only to classify perturbations, but also to dynamically regulate its sensitivity based on the history and goal.

Further, since the profile of the recent error signal has been successfully shown to be distinguishable, it could potentially be used in adaptive motor control policies. The profile could be used to trigger different appropriate behaviour protocols that handle unexpected events. A sharp peak associated with a push could trigger reorientation or stabilisation, sustained force could trigger compliance, obstacle contact could trigger a halt, while expected movement-related current errors could be attenuated.

### 4.3 Prediction-feedback balance

The neural network received a blended input of previous predictions and actual used current, weighted at 80% and 20%. This fixed weighting can be interpreted as analogous to a precision-like balance between model prediction and sensory evidence (Den Ouden et al., 2012; Adams et al., 2013), although precision was not estimated explicitly in the present architecture. The weighting was as a design choice to prevent error drift and was not systematically varied. Its effect on the morphology of error profiles and downstream classification performance remains an open question, and future work should evaluate the sensitivity of classification performance to this parameter under conditions where perturbation scenarios can be exactly replicated. Further, in a more biologically realistic architecture, the blend could be dynamically adjusted to favour internal predictions in noisy environments or to favour sensory feedback during novel interactions.

However, the goal need not be solely to minimise prediction error by maximising prediction accuracy. Our experiment shows how prediction errors can be utilised in classification, where the profile of the error signal informs the system about the types of perturbation. This can help distinguish self-generated movement from externally caused disruption of a planned trajectory—a core aspect of sensory attenuation. Imperfect predictions can therefore be a resource for the system rather than just a target for minimisation. Ideally, this would still include a form of online learning that can adapt to different contexts where interaction with external objects places different loads on the joints. In our experiment, we used offline learning to test how far proprioceptive information can be used in a controlled setting.

### 4.4 Limitations and future work

The present study should be interpreted as a proof-of-principle demonstration in a deliberately constrained setting. The experiments were conducted on a single two-degrees-of-freedom robotic platform, and the interaction set consisted of a limited number of well-separated conditions. Manual perturbations were applied by a single operator and from a consistent direction, while obstacle interactions were tested during a fixed 30° movement. Consequently, the current results do not establish that the observed prediction-error profiles generalise across operators, perturbation directions, movement contexts, or more heterogeneous physical interactions.

A broader evaluation could vary perturbation direction, onset time, force magnitude, obstacle location, and movement trajectory, and could include both transient and sustained perturbations during ongoing movement as well as an unperturbed static condition. Multiple operators, repeated recording sessions, or an automated and calibrated force source would also help determine whether the observed profiles reflect general properties of the interaction categories rather than idiosyncratic characteristics of the experimental procedure. The aim of the present experiment was not to exhaustively sample the continuous space of possible physical interactions, but to test whether qualitatively different interaction conditions could produce distinguishable prediction-error dynamics. Future work should therefore focus on systematic generalisation tests rather than simply increasing the number of arbitrarily discretised conditions.

The feature representation used for classification also reflects the structure of the present experiment. Features such as decay time, early-to-late error ratio, and phase-binned means were chosen to capture transient, persistent, and temporally localised deviations. Although these descriptors were useful for the tested conditions, it remains unclear whether the same representation would separate less distinct interaction types or previously unseen categories. More general representations, including direct classification of the error time series or learned temporal embeddings, could reduce dependence on hand-crafted features.

Scalability to higher-DOF systems also remains to be established. One possible extension would be to assign local predictors to individual joints and combine them within a hierarchical architecture. This is in line with the hierarchical prediction-minimisation schemas previously suggested in active inference (Adams et al., 2013). Higher-order predictive controllers can then handle more abstract predictions related to motor planning and goal trajectory. However, in more complex robots, actuator loads are coupled across joints, and self-collision, gravity, inertia, and interaction forces may produce overlapping current signatures. A per-joint predictor may therefore become insufficient once the expected load of one joint depends strongly on the state of other joints. Possible extensions include predictors that model coupled kinematic chains, hierarchical predictive models, or latent representations that capture interactions between multiple joints. These possibilities are directions for future investigation rather than demonstrated properties of our experiment.

### 4.5 Conclusions

The dynamics of load-related prediction error are not only a result of imperfect control but carry information about the environment. By using a windowed multilayered perceptron to predict servo current from proprioceptive signals, the error signal could be used for perturbation classification. Further, giving a temporal context of three lags allowed the network to infer local movement dynamics. This illustrates the power of internal generative models for learning the body's implicit physics without specialised inertial sensors. These results motivate future investigation of whether such internal models can be extended to hierarchical and higher-DOF architectures for learning body-environment dynamics.

## Data Availability

The datasets presented in this study can be found in online repositories. The names of the repository/repositories and accession number(s) can be found below: https://github.com/pierreklintefors/ikaros/tree/master/Source/Modules/RobotModules/CompliantRobot.
