# Supplementary material for: Temporal signatures of prediction error reveal external forces on an embodied robot
Source: Front Neurorobot. 2026 Oct 14;20:1918275. doi: 10.3389/fnbot.2026.1918275 (PMC13713828; doi:10.3389/fnbot.2026.1918275)
Supplement: Supplementary file 1 [file Data_Sheet_1.pdf]

Appendix: Feature Extraction for Perturbation Experiment

To capture the the geometric, temporal, and dynamic structures of the prediction error profiles across experimental trials, a set of hand-crafted features was extracted. For all subsequent mathematical formulations, let $e(t)$ represent the discrete error signal vector of length N, where $t \in \{0, 1, \ldots, N-1\}$.

Phase Bin Means

This feature partitions the total trial duration into B equal-length, non-overlapping temporal windows and computes the mean absolute error within each window. This constructs a discrete, low-resolution profile of the error's evolution across the trial. For the k-th bin (where $k \in \{0, \ldots, B-1\}$) spanning the sample indices $t \in [t_{\text{start}}, t_{\text{end}}]$, the feature value μ_k is defined as:

$$\mu_k = \frac{1}{t_{\text{end}} - t_{\text{start}} + 1} \sum_{t=t_{\text{start}}}^{t_{\text{end}}} |e(t)| \tag{1}$$

Early-to-Late Ratio

This metric quantifies the balance of error magnitude between the initial and final phases of the movement by computing the ratio of their respective mean absolute errors. Values $R_{EL} \gg 1$ indicate rapid, front-loaded transients; values $R_{EL} \approx 1$ denote sustained errors; and values $R_{EL} < 1$ indicate late-stage deviations or obstacle-induced perturbations. Given a fractional parameter $\alpha \in (0, 0.5)$, let $N_{\text{early}} = \lfloor \alpha N \rfloor$ and $N_{\text{late}} = \lfloor (1-\alpha) N \rfloor$:

$$R_{EL} = \frac{\frac{1}{N_{\text{early}}} \sum_{t=0}^{N_{\text{early}}-1} |e(t)|}{\left(\frac{1}{N - N_{\text{late}}} \sum_{t=N_{\text{late}}}^{N-1} |e(t)| \right) + \epsilon} \tag{2}$$

where $\epsilon = 10^{-9}$ is a regularization constant introduced to prevent division-by-zero errors during near-zero late phases.

Time-to-Peak

This feature identifies the normalized temporal location along the interval $[0, 1]$ where the error reaches its maximum absolute magnitude. This effectively differentiates early-stage impact dynamics from late-stage kinematic deviations during target approach.

$$T_{\text{peak}} = \frac{1}{N} \arg\max_t |e(t)| \tag{3}$$

Half-Decay Time

This feature measures the normalized time required for the absolute error to decay below 50% of its peak value following the moment of peak impact. If the error remains above this threshold for the remainder of the trial, the feature returns 1.0, signaling a sustained, uncorrected perturbation. Let $t_{\text{peak}} = \arg\max_t |e(t)|$. Defining the post-peak index offset τ^* as:

$$\tau^* = \min\{\tau \in \mathbb{N}_0 \mid |e(t_{\text{peak}} + \tau)| \leq 0.5 \cdot |e(t_{\text{peak}})|\} \tag{4}$$

The normalized half-decay time T_{decay} is given by:

$$T_{\text{decay}} = \begin{cases} \frac{\tau^*}{N} & \text{if } \tau^* \text{ exists} \\ 1.0 & \text{otherwise} \end{cases} \tag{5}$$

Lag-1 Autocorrelation

This feature computes Pearson's linear correlation coefficient between adjacent time steps ($e(t)$ and $e(t+1)$) to measure the temporal smoothness and macro-structural persistence of the error. Coefficients near 1.0 represent highly structured, continuous deviations, whereas near-zero or negative values indicate highly oscillatory, transient, or noisy control corrections. Let $\bar{X}$ be the mean of the sub-vector $e(0 \ldots N-2)$ and $\bar{Y}$ be the mean of $e(1 \ldots N-1)$:

$$r_{\text{lag1}} = \frac{\sum_{t=0}^{N-2}(e(t) - \bar{X})(e(t+1) - \bar{Y})}{\sqrt{\sum_{t=0}^{N-2}(e(t) - \bar{X})^2 \sum_{t=0}^{N-2}(e(t+1) - \bar{Y})^2}} \tag{6}$$

Temporal Center of Mass

By treating the absolute error trajectory over time as a spatial distribution of mass, this feature locates its normalized balancing point along the interval $[0, 1]$. It provides a continuous scalar metric indicating whether the kinematic profile is front-, mid-, or back-loaded.

$$\text{CoM}_{\text{temp}} = \frac{\sum_{t=0}^{N-1} t \cdot |e(t)|}{N \cdot \sum_{t=0}^{N-1} |e(t)|} \tag{7}$$

DCT Shape Coefficients

This feature transforms the peak-normalized error profile into the frequency domain via an orthonormal Type-II Discrete Cosine Transform (DCT-II). The resulting low-frequency coefficients compactly parametrise broad geometric shapes: c_1 characterizes the linear trend, while c_2 captures continuous parabolic curvature. Let $\hat{e}(t) = \frac{e(t)}{\max_t |e(t)| + \epsilon}$. The k-th coefficient is defined as:

$$c_k = \sqrt{\frac{2}{N}} w_k \sum_{t=0}^{N-1} \hat{e}(t) \cos\left(\frac{\pi k(2t+1)}{2N}\right), \quad \text{for } k \in \{1, \ldots, n_{\text{coeff}}\} \tag{8}$$

where the scaling factor $w_k = \frac{1}{\sqrt{2}}$ for $k = 0$ (omitted from the final feature vector as it represents the redundant mean profile) and $w_k = 1$ for $k \geq 1$.

Sign Consistency

This metric evaluates the proportion of the trial's duration during which the error preserves the same directional orientation as the dominant peak error. Values near 1.0 specify a highly unidirectional biomechanical or external load, while values near 0.5 imply zero-crossing oscillations or dynamic instability around the target trajectory. Let $s_{\text{dom}} = \text{sgn}\left(e(\arg\max_t |e(t)|)\right)$ and $\mathbb{I}$ represent the indicator function:

$$S_{\text{const}} = \frac{1}{N} \sum_{t=0}^{N-1} \mathbb{I}\Big(\text{sgn}(e(t)) = s_{\text{dom}}\Big) \tag{9}$$
